# The use of a testimonial video featuring diverse ADRC participants to change research attitudes in the elderly community

**DOI:** 10.1002/alz.089844

**Published:** 2025-01-09

**Authors:** Margaret Sewell, Judith A. Neugroschl, Mari Umpierre, Shehan Chin, Jade Nunez, Scarline Martinez, Paulette Fernandez, Vielka Inoa, Mary Sano

**Affiliations:** ^1^ Icahn School of Medicine at Mount Sinai, New York, NY USA; ^2^ Icahn school of medicine, New York, NY USA; ^3^ Brown University, Providence, RI USA; ^4^ City University of New York, New York, NY USA; ^5^ Stetson University, Deland, FL USA

## Abstract

**Background:**

Black, Latinx, and Asian community elders remain underrepresented in dementia research. This study explored whether a video created to promote diversity in research by featuring underrepresented ADRC participants would change attitudes about research generally (e.g., that it is safe, that it will find cures), as well as change attitudes about participation in specific types of research (e.g., involving imaging or blood draws).

**Method:**

We created a testimonial format 4‐minute video featuring three ADRC participants (Chinese, Black, Hispanic). They discussed the importance of diversity in research and the vignettes illustrated participation in blood draws, imaging, and cognitive testing. Attitudes were measured by the 7‐item Research Attitude Questionnaire, before and after watching the video. Additionally, four Likert‐style questions were asked regarding the importance of diversity participation in research and willingness to participate in studies involving imaging, blood draws, or cognitive testing.

**Result:**

The video was presented at 6 senior centers in NYC with 91 attendees (age 52‐90). 79% female, 59% Black, 39% Hispanic/Latino, and 8% other. Regarding education, 30% < high school, 35% high school graduates, and 35% ≥ some college. Spanish was the primary language for 44%. The RAQ item “*Participating in medical research is generally safe*” had the greatest pre/post video delta, with a 14% increase (62% to 76%) endorsing Agree/Strongly Agree. Other RAQ items that were relevant to the video vignettes (e.g., “*We all have some responsibility to help others by volunteering in research”* had a more modest (5% to 7%) increase in Agree/Strongly Agree. Concerning the additional questions, the greatest increases were seen concerning the need for diversity (66% to 81% Agree/Strongly Agree), and willingness to consider imaging studies (52% to 62%). The greatest trepidation was regarding participating in studies with blood draws (46% initially endorsed Agree/Strongly Agree) which only increased to 49% after the video.

**Conclusion:**

We demonstrate here how testimonial videos with peers may impact interest in research. Future work will explore how improved attitudes toward research may increase research enrollment.

